# The Engineering Side of Hospital Work

**Published:** 1905-03-25

**Authors:** 


### THE ENGINEERING SIDE OF HOSPITAL WORK.

### VII.—DISINFECTORS AND STERILISERS.

(*Continued from page* 454)

#### Sterilisers.

There are various forms of sterilisers, arranged for various duties, such as sterilising surgical instruments after use, and these are fitted in various ways. Generally the apparatus consists of a trough containing either water or a sterilising solution, maintained at a certain temperature by any convenient means. Steam is frequently used, gas is also employed, and electricity has been made use of, but owing to the great cost of the heat developed by the electric current, as against the cost of generating the same quantity by gas or steam, the application has not met with favour. It is a very convenient method, and that is all that can be said about it at present. Of the other two methods, steam is undoubtedly the best, if the source of steam is not too far away. Unfortunately steam is subject to considerable losses by condensation, when it is transmitted over long distances. The pipes in which it is carried, radiate heat at every foot, and the heat so radiated is taken from the steam the pipe contains, the result being that the pressure of the steam is reduced, and some of it is constantly condensing, this meaning considerable waste of the coal used in the furnace of the boiler where the steam is generated. Gas is more economical, under almost all conditions, but it is often objectionable. The question of power transmission by steam will be dealt with in a later article. With hospitals spread out as they are, power transmission and heat transmission are very important questions.

But there is another form of steriliser that is of great importance, that may be looked upon as an adjunct to the disinfector, in which linen that has been soiled by infected patients, in cases of typhoid and others, is dealt with quickly and efficiently, and at less cost than by means of the disinfector. The rotary steriliser, as it is called, is really a rotary washing machine. It consists of a cylinder, sometimes built into the wall dividing the two compartments used for disinfecting, very much as the disinfector itself is, but the arrangement is much smaller. It has a door on each side of the partition, the clothes being put in on the infected side and removed on the pure side. The cylinder is given a rotary motion, the direction being reversed frequently. The clothes are first put in the apparatus, cold water run in, the cylinder rotated for a certain time till the clothes are rinsed, and the rinsing water run off into a vessel under the apparatus. Steam is then passed into the cylinder, the apparatus worked again for a certain time, the steam being allowed to get up to 15 lb. per square inch, gradually cooled, the clothes taken out and dried. One of these apparatus, made by Messrs. Manlove and Alliott and Co., is in use at

Middlesex Hospital. The machine is driven by an electric motor capable of furnishing 2 horse-power, and the reversals in the direction of rotation are arranged in a very ingenious manner. The shaft of the motor gears with the shaft of the cylinder, turning it in the usual way. From the shaft of the cylinder a chain passes to a revolving two-part commutator or reversing switch, which at a certain time, after the cylinder has made a certain number of revolutions, reverses the direction of the current in the motor, the direction of revolution of the motor and of the cylinder reversing in consequence. The arrangement appears to work very well indeed, there being very little wear at the reversing commutator and at the commutator of the motor.

The rinsing water from the steriliser is subjected to steam at a pressure of 15 lb. per square inch, thoroughly killing any bacilli that may be present.

Disinfectors in Use in London Hospitals.

Of the hospitals the writer visited, Westminster and King's College, as mentioned, have hot-air disinfecting apparatus, the Royal Free Hospital has none, Charing Cross has none, St. George's has an old pattern Washington Lyon machine, St. Bartholomew's has a Washington Lyon with a small boiler specially arranged to supply it with steam, and the others, St. Thomas's, St. Mary's, Middlesex, London, University, and Guy's, have Messrs. Manlove, Alliott and Co.'s improved arrangement with the vacuum attachment.

It should perhaps be mentioned that steam for the disinfectors is taken from the nearest boilers, as convenient. The quantity of steam required is not large, and it is not difficult to arrange that disinfecting goes on when there is not a large call for steam for other purposes. It is a very wasteful plan to generate steam specially for the disinfector, unless it is inconvenient to carry steam pipes to the disinfector or in those few cases where the waste by condensation would be greater than the waste due to the small boiler. Small boilers always consume much more coal for a given quantity of steam than large boilers, and in addition there is always a considerable waste of coal in getting up steam, and when the steam is allowed to run down, as when the disinfecting is finished.

